# Acute Pain in Children with Chronic Musculoskeletal Pain: A Prospective Controlled Study of Intensive Interdisciplinary Treatment

**DOI:** 10.3390/children12101357

**Published:** 2025-10-09

**Authors:** Rebecca Wells, Mackenzie McGill, Sabrina Gmuca, Ashika Mani, David D. Sherry

**Affiliations:** 1 Department of Psychiatry and Behavioral Sciences, University of California San Francisco, San Francisco, CA 94107, USA; rebecca.wells@ucsf.edu; 2 Department of Pediatrics, Division of Rheumatology, Children’s Hospital of Philadelphia, Philadelphia, PA 19104, USA; gmucas@chop.edu (S.G.); sherry@chop.edu (D.D.S.); 3 Center for Pediatric Clinical Effectiveness, Children’s Hospital of Philadelphia, Philadelphia, PA 19146, USA; 4 PolicyLab, Children’s Hospital of Philadelphia, Philadelphia, PA 19146, USA; 5 Biostatistics Analysis Center (BAC), University of Pennsylvania, Philadelphia, PA 19104, USA; ashika.mani@pennmedicine.upenn.edu

**Keywords:** acute pain, chronic pain, pediatrics, pain threshold, pain tolerance

## Abstract

**Objectives**: Chronic pain corresponds to hypersensitivity to painful stimuli; however, its relation to acute pain sensitivity in children is poorly understood. We explored this relationship by comparing acute and chronic pain measures, along with related factors, in children with chronic pain syndromes versus controls, before and after therapeutic intervention. **Methods**: This prospective controlled cohort study involved 57 children with chronic pain undergoing intensive interdisciplinary pain treatment in a hospital-based pain rehabilitation program and 50 controls. Participants, aged 7–18, were tested using a cold pressor task (CPT) at admission, discharge, and first follow-up visit. Data on sleep, anxiety, psychological distress, functional impairment, and pain were collected. **Results**: Significant differences were found between control and treatment groups in average pain threshold (*p* < 0.001), pain tolerance (*p* = 0.035), sleep visual analog scale (VAS) (*p* < 0.001), functional disability inventory (*p* < 0.001), patient reported outcomes information system anxiety assessment tool (*p* < 0.001), general anxiety disorder 7-item scale (*p* < 0.001), pain VAS (*p* < 0.001) and total brief symptom inventory (BSI) (*p* < 0.001) scores at admission with children with chronic pain scoring worse on all measures save the pain VAS during the CPT. After treatment and at follow-up, function and mental health measures improved but not acute pain threshold. **Conclusions**: At treatment completion, function and mental health significantly improved but acute pain threshold and sleep quality were unchanged. These findings suggest that while chronic pain treatment improves overall function and mental health, acute pain thresholds may not be a suitable indicator for evaluating the efficacy of interventions.

## 1. Introduction

Pediatric chronic pain is a debilitating condition that affects many children’s daily function, yet the underlying mediator remains unknown and measurement remains highly subjective [1,2]. Existing theories suggest that chronic pain may stem from an abnormal deficiency of pain inhibition or a symptom of disordered sleep, which are both characteristics of children with chronic pain alongside noted increases in anxiety and functional impairment in activities of daily living [3,4,5]. While pain is difficult to reliably and ethically measure, the experience of acute pain can be experimentally characterized by pain threshold, the initial point at which pain is felt, as well as pain tolerance, the amount of pain considered endurable. An inhibitory deficit could manifest as a lower pain tolerance, lower pain threshold, or both; these metrics can be reliably and ethically demonstrated via the cold pressor task (CPT).

There have been mixed findings regarding the pain tolerance of children with chronic pain. One CPT study showed that children with juvenile idiopathic arthritis have lower pain tolerance compared to controls [6]. Conversely, another CPT study showed no significant difference in pain tolerance between children with chronic pain and healthy controls [7]. These mixed findings suggest the need for further investigation of pain tolerance.

In contrast, research on pain threshold in adult chronic pain patients has been relatively conclusive, finding a lower pain threshold in patients with chronic pain. One study reported that adult patients with fibromyalgia syndrome (FMS), or amplified musculoskeletal pain syndrome (AMPS), had a lower pain threshold for painful experimental stimuli involving heat, cold, and pressure than controls [8]. Another, using CPT to evoke an acute pain response, confirmed this finding in adult FMS patients [9].

Additionally, poor sleep quality has been associated with chronic pain in adolescents [10]. Poor sleep quality has been shown to improve significantly in adolescents after intensive interdisciplinary pain treatment (IIPT) consisting of rigorous physical and occupational therapy [11].

Aspects of the pain experience are crucial outcome domains for evaluating treatments for pediatric chronic pain [12]. This study aims to assess acute pain thresholds and tolerance before and after IIPT in a hospital-based pain rehabilitation program. The program includes daily 1:1 physical therapy (PT) and occupational therapy (OT), individual and group cognitive-behavioral therapy (CBT), parent therapy sessions, and art and music therapy. While this biopsychosocial model-based program has been shown to be effective in alleviating chronic pain symptoms, improving function, and enhancing sleep quality, its impact on acute pain remains unclear [13,14,15,16,17].

We hypothesized that children with more severe chronic pain will be hypersensitive to acute pain, and therefore demonstrate lower acute pain thresholds and tolerances than healthy controls, as measured via the CPT. Additionally, we suspect that these children will experience more disordered sleep. We expect that improvements in chronic pain, evident upon discharge and follow-up, will correspond to higher acute pain thresholds and tolerance, as well as improved reported sleep quality.

## 2. Materials and Methods

### 2.1. Study Design, Population, and Setting

This was prospective controlled cohort study utilizing a convenience sample pre- and post-test study for subjects enrolled in a hospital-based pain rehabilitation program that delivers IIPT and a one-time comparison for control subjects. Evaluators were not blind to the groups. The study received Institutional Review Board approval from The Children’s Hospital of Philadelphia and was conducted between September 2015 and February 2023. Active participants included patients 7–18 years old, being admitted to the IIPT program who were evaluated in the outpatient Rheumatology clinic and diagnosed with amplified musculoskeletal pain syndrome (defined as disproportionate pain and disability to any inciting event) by the treating medical provider (physician and/or advance practice provider) [18]. Exclusion criteria included: (1) those unable to comply with protocol requirements; (2) subjects who have a history of fractures within the past 3 years in the limb to be used in testing, history of seizures, history of frostbite, history of seizures, history of cardiovascular disease, chronic musculoskeletal pain (CMP) specific to the upper extremities, history of complex regional pain syndrome (CRPS), or history of Raynaud phenomenon (decreased blood flow in the extremities); (3) parents or subjects unwilling or unable to provide consent for participation. This study received local institutional review board approval. Control participants included males and females 7–18 years old with no diagnosis of amplified pain and able to provide consent/assent.

### 2.2. Recruitment

*Active Subjects*: Active subjects were pre-screened based on IIPT program weekly admissions and discharges. During the IIPT, patients undergo daily 1:1 physical therapy (PT), occupational therapy (OT), as well as individual and group cognitive behavioral therapy (CBT)-based interventions. Creative arts therapy (art and group music therapy) was also incorporated into treatment. Duration of treatment was tailored to each individual patient’s needs, and typical length of stay in the program was 2–3 weeks. Patients deemed potentially eligible were then approached upon admission and explained the purpose and procedures of the study to gauge interest. If interested, the patient and parent/guardian were consented at that time. Active participants received USD 5 at the first study visit (admission), and then another USD 5 at the final visit (first follow-up after discharge, approximately 2–3 months post-discharge) via pre-paid gift card.

*Control Subjects*: Controls subjects were recruited via convenience sample at the general rheumatology and subspecialty pediatric rheumatology pain clinics to match the basic demographics of the active subjects. We approached children in rheumatology clinic with benign diagnoses (e.g., ANA positive, hypermobility syndrome) and no diagnosis of amplified musculoskeletal pain for participation, as well as unaffected siblings in pain clinic. We also advertised the study through local community boards and various outreach methods, including social media and partnerships with community organizations. Potential control participants were identified and reached out to about participating in the study. Those interested would be scheduled to provide informed consent and visit in-person. Control participants were compensated USD 10 via pre-paid gift card for their time and completion of study procedures.

All subjects were consented using the local IRB-approved informed consent form as guidance and all questions were answered in accordance with IRB regulations.

### 2.3. Questionnaires

*Sleep Visual Analog Scale (Sleep VAS)*

Participants were asked to rate their subjective sleep quality over the past week and day as a number from 0 to 100 by demarcating each rating on a line with ends marked “0” and “100”. Higher scores indicated worse sleep quality [19].

*Pain Visual Analog Scale (Pain VAS)*

Participants were asked to rate their subjective current pain before completing the CPT and while their hand was in the cold water as a number from 0 to 100 by demarcating each rating on a line with ends marked “0” and “100”, with “0” indicating no pain and “100” indicating the worst pain possible [20].

*Functional Disability Inventory (FDI)*

This is a 15-item measure of pain related disability [21]. It assesses the impact of adolescents’ physical health on physical and psychosocial functioning (i.e., walking up stairs, performing chores, being at school all day, performing different activities, etc.) in the past 2 weeks using a Likert-scale (0 = “No Trouble” to 4 = “Impossible”), with higher scores indicating greater difficulty functioning (no/minimal (0–12), mild (13–20), moderate (21–29), and severe (≥30)) [22]. Total scores are computed by summing the rating for each of the 15 items on the FDI. The FDI has demonstrated adequate psychometric properties with youth ages 8–18 years with CMP. The FDI has been found to have high internal consistency, moderate to high test–retest reliability, and good predictive validity [23].

*Brief Symptom Inventory (BSI)*

Brief symptom inventory (BSI) consists of 53 items which assess psychological distress across domains in the past week [24]. These items cover nine symptom dimensions: somatization, obsession-compulsion, interpersonal sensitivity, depression, anxiety, hostility, phobic anxiety, paranoid ideation, and psychoticism. Respondents rank each item (e.g., “feeling easily annoyed or irritated”, “suddenly scared for no reason”, feeling that people are unfriendly or dislike you”, “trouble getting your breath”, “feeling very self-conscious with others”, “feelings of guilt”, etc.) on a 5-point scale ranging from 0 (not at all) to 4 (extremely). Rankings depict the intensity of distress during the past seven days. We calculated the global severity index (GSI) using the sums for the nine symptom dimensions plus the four additional items not included in any of the dimension scores, and dividing by the total number of items to which the individual responded. The global indices measure current or past level of symptomatology, intensity of symptoms, and number of reported symptoms, respectively. T-scores of 63 and above should be considered clinically significant distress. The BSI has been validated as an effective tool for studying adolescents aged 13–17 years. The BSI has been found to have good internal consistency reliability, test–retest reliability, and validity [24,25].

*General Anxiety Disorder 7-Item Scale (GAD-7)*

The GAD-7 is a 7-item scale which screens for trait anxiety symptoms over the last two weeks. For each symptom queried based on the *DSM-IV* (i.e., feeling nervous, worrying, trouble relaxing, restlessness, etc.), it provides the following response options: “not at all,” “several days,” “over half the days”, and “nearly every day” and these are scored, respectively, as 0, 1, 2, or 3. Total scores for the seven items were taken, with 0–4 defined as minimal anxiety, 5–9 as mild anxiety, 10–14 as moderate anxiety, and ≥15 as severe anxiety. GAD-7 has demonstrated excellent internal consistency, good test–retest reliability, as well as good criterion, construct, factorial, and procedural validity [26,27,28].

*PROMIS (Patient Reported Outcomes Information System) Anxiety Assessment Tool-Short Form*

This self-report measure evaluates pediatric anxiety severity. Participants were asked to respond, using a Likert-scale from 1 = “Never” to 5 = “always” to gauge how often the participant felt fearful, found it hard to focus on anything other than anxiety, felt that their worries overwhelmed them, felt uneasy, felt nervous, felt that they needed help for their anxiety, felt anxious, and felt tense in the past seven days. The raw score was converted to a standard score (t-score) with a conversion chart. A higher t-score represents more perceived anxiety. Internal consistency of this short form has been demonstrated as excellent [29].

### 2.4. Cold Pressor Test

**Figure 1:**
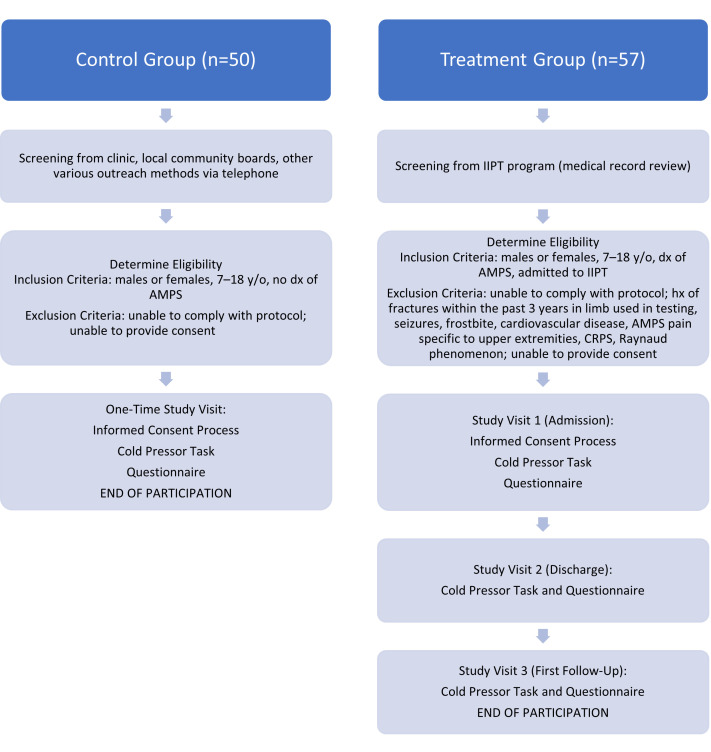
Timepoints for data collection—control vs. treatment groups. (y/o = years old, dx = diagnosis, AMPS = amplified musculoskeletal pain syndrome, IIPT = intensive interdisciplinary pain treatment, hx = history).

This study uses the cold pressor test, which has been validated for use in children as a tool for measuring acute pain threshold and tolerance [30,31]. All subjects were asked to leave their hand in water measured at 36 (+/−1) degrees Celsius for one minute. The baseline temperature of the finger was recorded. Next, the subject was timed while submerging his/her hand in cold water (held as close as possible to 10 (+/−1) degrees Celsius) for as long as possible, starting with their left hand, regardless of hand dominance. Subjects reported the point at which pain is first felt and the time from hand immersion to report of pain was recorded as his/her pain threshold. Subjects could remove his/her hand from the water bath at any point. The test was stopped at a maximum of three minutes. Total duration of hand immersion was recorded as his/her pain tolerance. This task was completed according to the guidelines of cold pressor use with children [30]. Active participants were tested with questionnaires and the cold pressor task procedure at entry to and discharge from IIPT program, and then at his/her first follow-up visit post-discharge. Control participants were tested once, at the convenience of the participant and parent/guardian, and the collected values were used as a normal baseline. All subjects completed the CPT in an outpatient clinical setting following this validated protocol, as shown in Figure 1.

### 2.5. Data Collection

Patient demographics and clinical characteristics were abstracted from the electronic medical record in Research Electronic Data Capture (RedCap) and merged with data from the clinic’s existing patient registry. Questionnaires were answered and stored in REDCap.

### 2.6. Data Analysis

Baseline and demographic characteristics were summarized by standard descriptive summaries (e.g., means and standard deviations for continuous variables such as age, percentages for categorical variables such as gender). We compared the demographic and baseline characteristics between controls and those in the treatment group for continuous variables using 2-sample *t*-tests. For categorical variables, we utilized chi-square tests of independence.

The primary analysis included all subjects meeting inclusion and exclusion criteria and completing Visit 1 (see Figure 1). The primary endpoint was the change in acute pain threshold between Visit 1 and discharge (+/−1 day) from the IIPT program. Secondary endpoints included the pre- vs post-intervention change in pain tolerance, sleep VAS score, pain VAS score, FDI score, BSI score, GAD-7 score, and PROMIS anxiety score. Additionally, data from controls were used as a comparison for values on all data points of subjects before and after treatment.

A multivariate linear mixed-effects model was used to measure the association between demographics information, questionnaire results, and both pain threshold and pain tolerance. We utilized the lmer command from the *lmerTest* package in R. A random subject effect was included in our model, with age, gender, visit type (admission as the reference group), sleep VAS week, sleep VAS day, FDI score, PROMIS score, GAD score, and total BSI included as fixed effects. A two-sided *p*-value < 0.05 was indicative of statistical significance for all tests. All statistical analyses were conducted using R version 4.1.2 (R Project for Statistical Computing).

### 2.7. Sample Size and Power

Based on previous CPT studies looking at adult FMS subjects as compared to controls, and aiming to obtain 90% power, a sample size of 46 per group was needed to reach significance for threshold, and 31 for tolerance.

## 3. Results

A total of 50 control participants and 57 treatment participants were enrolled in this study, with 59.6% of treatment participants completing all study timepoints. The only reason for incomplete participation was loss to follow-up, in which case their data was included for relevant timepoints. Table 1 provides patient demographics and clinical characteristics. For the control group, 25 (50%) were female, 21 (42.0%) were male, and 4 (8.0%) did not provide their sex. In the treatment group, 40 (70%) were female, and 17 (30%) were male. The average age was 13 years (SD 3.17) for the control group and 14.6 years (SD 2.57) for the treatment group. There were no statistically significant differences in age or sex between the control and treatment groups.

When comparing control subjects to those in the treatment group at admission, all measured variables except the pain VAS during the CPT were significant (Table 2). Children with chronic pain at admission demonstrate lower acute pain thresholds (*p* < 0.001) and tolerances (*p* < 0.05). Additionally, children with more severe chronic pain at admission have higher sleep VAS, indicating worse sleep quality, on both a weekly (*p* < 0.001) and daily (*p* < 0.001) basis. At baseline, the treatment group had a significantly lower pain threshold, and significantly higher FDI, and pain VAS scores compared to the control group (all *p* < 0.001). This remained true following treatment at both discharge (pain threshold *p* < 0.003; FDI and pain VAS *p* < 0.001) and follow-up (pain threshold and pain VAS *p* < 0.001, FDI *p* = 0.002). Sleep VAS week and day, PROMIS, and GAD-7 remained significant as compared to the control group until follow-up (at admission all *p* < 0.001; at discharge sleep VAS week *p* < 0.001, sleep VAS day *p* = 0.011, PROMIS *p* = 0.001, and GAD-7 *p* = 0.047). The difference between controls and subjects on pain tolerance (*p* = 0.035) and BSI (*p* < 0.001) was only significant at admission.

Children with chronic pain demonstrated a statistically significant difference on a variety of measures between admission and discharge from the IIPT program (Table 3). At discharge, FDI (*p* < 0.001), PROMIS (*p* = 0.013), and BSI (*p* = 0.005) scores both significantly decreased. However, pain threshold, pain tolerance, weekly and daily sleep quality, GAD-7, and pain VAS describing both pain related to CPT and pain before CPT, did not significantly differ. When looking at the difference between admission and first follow-up post-treatment, we see the sustained improvement in FDI (*p* < 0.001), PROMIS (*p* < 0.001), and BSI (*p* < 0.001), and additional improvements in GAD (*p* < 0.001), and pain VAS before CPT (*p* < 0.001) scores. Weekly and daily sleep VAS scores and pain VAS related to CPT were not significant across all timepoints.

In the linear mixed-effects model, with average pain threshold as the outcome, age was a significant predictor when controlling for the other variables in the model (Table 4). As age increased, so did the pain threshold (*p* = 0.021). As FDI score increased, the average pain threshold increased (*p* = 0.04). For each unit increase in the pain VAS score (indicating greater recent level of chronic pain), average pain tolerance significantly decreased (*p* = 0.02) and pain threshold slightly decreased, but this was not statistically significant (*p* > 0.05). There was no significant difference in average pain threshold or pain tolerance between the different measurement times.

## 4. Discussion

Pain is a subjective experience, and one that ethics dissuade researchers from experimentally inducing. However, cold pressor was evaluated as a reliable and ethically responsible measure of pain in children specifically [32,33]. While previous studies have demonstrated that patients with chronic pain syndromes have lower acute pain thresholds, more investigation is needed to determine the relationship of chronic pain to the experience of acute pain tolerance, as well as how the experience of acute pain (as measured by acute pain threshold and tolerance) might vary as chronic pain is treated and improves.

The goal of our study was to determine the relationship between the acute and chronic pain experiences and sleep quality in children with chronic pain, and how these factors may or may not change following participation in the IIPT program. In addition to re-demonstrating efficacy of the IIPT program, as indicated by improved functional and psychological measures following treatment, we found that while it does not reach the level of statistical significance, sleep quality trends toward improvement following treatment.

Our results reveal that patients with chronic pain showed significantly lower average pain tolerance and threshold compared to controls, indicating greater sensitivity to acute pain stimuli. Within the treatment group, these measures did not significantly change after treatment, despite clinical improvements in several other areas. In addition to lower pain threshold and tolerance, patients with chronic pain exhibit poorer sleep quality, more functional disability, and greater anxiety, psychological distress, and degree of pain experienced compared to controls. Significant improvements were observed in functional and symptom-related measures (FDI, PROMIS, GAD-7, total BSI, and pain VAS) from admission to follow-up, re-demonstrating the known benefit of IIPT for the management of pediatric chronic pain [15].

Despite these improvements, pain threshold and tolerance remained stable throughout the treatment and follow-up periods. This stability underscores that, contrary to what we hypothesized, acute pain threshold and tolerance are likely not a good proxy for determining treatment effects. However, they may take time to improve after treatment, or they may be more static characteristics of this population and not readily malleable. It is possible that the stability of acute pain metrics before and after treatment reflects an ongoing difficulty with coping with discomfort that is not resolved by the time of treatment follow-up or a conditioned expectation of what a painful experience may feel like based on their history of sensitivity to pain. While most of these outcomes were already significantly improved by the time of discharge, the improvement in sleep quality was not observed.

There were several limitations of this study that need to be addressed. We did not collect participant information on race or ethnicity. This limits the generalizability of our findings and prevents this study from potentially measuring disparities in access to or efficacy of care. While not statistically significant, there were also differences in the age and sex of the patients in the control vs. the treatment group. Better matching of controls to our clinical sample would enable better generalizability of our results and remove these as possible confounding factors, though this seems unlikely based on the results of the linear mixed-effects model. Additionally, there was variability in follow-up scheduling among patients, with the average follow-up appointment occurring approximately 1 to 2 months after discharge from the program. However, this timing was not consistent for all patients—some attended sooner, some later, and some did not attend at all. Because there are uneven sample sizes between admission and discharge groups due to attrition, we are unable to calculate a paired t-test so that individual differences between participants can be eliminated. This limits our ability to control for individual differences and sampling errors. Furthermore, external factors such as concurrent medication use, the structured hospital environment, and varying levels of patient motivation may have influenced performance on the CPT independently of the treatment program. These factors were not systematically controlled for and may explain the lack of observed changes in CPT scores. Future research should consider accounting for the timing of follow-up visits and potentially only include patients who complete all study sessions. It may be beneficial to include a second follow-up visit or extend the follow-up period to better assess the durability of treatment benefits. Future studies should consider examining the impact of individual treatment components (e.g., physical therapy vs. psychological therapy) to better understand the contribution of each treatment component to patient outcomes.

Our findings indicate that acute pain tolerance and threshold are both significantly lower in the population with chronic pain as compared to controls at baseline, and that this difference persists following treatment, despite functional improvement. We therefore suggest that CPT measures of acute pain threshold and tolerance are likely not appropriate proxy measures to use while evaluating the course and treatment of chronic pain in children, but rather that treatment outcomes are likely better evaluated through functional and psychological measures.

## Figures and Tables

**Table 1:** Patient Demographics. (N = number of subjects, SD = standard deviation, Min = minimum, Max = maximum, * 4 controls did not self-identify a sex).

	Control—Baseline (N = 50)	Treatment—Admission (N = 57)	Treatment—Discharge (N = 51)	Treatment—Follow-Up (N = 34)	Overall (N = 192)	*p*-Values Control vs. Admission	*p*-Values Control vs. Discharge	*p*-Values Control vs. Follow-Up
**Age (years)**						0.006	0.016	0.063
Mean (SD)	13.0 (3.2)	14.6 (2.6)	14.4 (2.6)	14.2 (2.6)	14.0 (2.8)			
[Min, Max]	[7.2, 18.1]	[8.3, 17.9]	[8.3, 17.9]	[9.5, 17.9]	[7.2, 18.1]			
**Sex**						0.024	0.042	0.080
F	25 (50%)	40 (70%)	35 (69%)	24 (71%)	124 (65%)			
M	21 (42%) *	17 (30%)	16 (31%)	10 (29%)	64 (33%)			

**Table 2:** Comparison of clinical findings in controls vs. treatment group at admission, discharge, and follow-up. (N = number of subjects, SD = standard deviation, VAS = visual analog scale, FDI = functional disability inventory, PROMIS = patient reported outcomes information system anxiety assessment tool; GAD-7 = general anxiety disorder 7-item scale; BSI = brief symptom inventory; CPT = cold pressor test; * = statistically signficant).

	Control—Baseline (N = 50)	Treatment—Admission (N = 57)	Treatment—Discharge (N = 51)	Treatment –Follow-Up (N = 34)	*p*-Values Control vs. Admission	*p*-Values Control vs. Discharge	*p*-Values Control vs. Follow-Up
**Acute Pain**							
**Avg. Pain Threshold (seconds)**					<0.001 * (d = −0.8)	<0.003 * (d = −0.7)	<0.001 * (d = −0.9)
Mean ± SD (95% CI)	88.4 ± 67 (69.3, 107.4)	46.4 ± 39.7 (35.9, 56.9)	47.4 ± 51.1 (33.1, 61.8)	39.1 ± 36.2 (26.4, 51.7)			
**Avg. Pain Tolerance (seconds)**					0.035 * (d = −0.5)	0.069 (d = −0.5)	0.293 (d = −0.5)
Mean ± SD (95% CI)	121.8 ± 63.2 (103.8, 139.7)	90.4 ± 62.9 (73.7, 107.1)	91.5 ± 68.3 (72.3, 110.7)	96.4 ± 70.9 (71.7, 121.1)			
**Sleep Impairment**							
**Sleep VAS Week**					<0.001 * (d = 0.9)	<0.001 * (d = 0.9)	0.303 (d = 0.9)
Mean ± SD (95% CI)	20.1 ± 20.8 (14.2, 26)	43 ± 28.3 (35.1, 51)	41.6 ± 27.8 (33.5, 49.8)	29.2 ± 25.6 (19.8, 38.6)			
Missing	0 (0%)	6 (11%)	4 (8%)	3 (9%)			
**Sleep VAS Day**					<0.001 * (d = 0.8)	0.011 * (d = 0.6)	0.546 (d = 0.3)
Mean ± SD (95% CI)	17.5 ± 20.6 (11.6, 23.3)	38.8 ± 29.8 (30.9, 46.7)	32.3 ± 28.4 (24.2, 40.4)	24.9 ± 26.6 (15.3, 34.5)			
Missing			1 (2%)	2 (6%)			
**Functional Impairment**							
**FDI**					<0.001 * (d = 2.7)	<0.001 * (d = 1.5)	0.002 * (d = 1)
Mean ± SD (95% CI)	2.7 ± 3.9 (1.6, 3.8)	24.5 ± 10.6 (21.6, 27.3)	11.9 ± 7.9 (9.7, 14.2)	8.8 ± 8.8 (5.7, 12)			
Missing			1 (2%)	2 (6%)			
**Anxiety Symptomatology**							
**PROMIS**					<0.001 * (d = 1.3)	0.001 * (d = 0.7)	**0.063** (d = 0.6)
Mean ± SD (95% CI)	10.6 ± 4.8 (9.2, 11.9)	19.9 ± 8.9 (17.5, 22.3)	15.3 ± 7.5 (13.1, 17.4)	13.5 ± 5.9 (11.4, 15.7)			
Missing	0 (0%)	1 (2%)	1 (2%)	2 (6%)			
**GAD 7**					<0.001 * (d = 0.9)	0.047 * (d = 0.5)	1.000 (d = 0.2)
Mean ± SD (95% CI)	9.2 ± 4 (8, 10.3)	13.7 ± 5.7 (12.2, 15.2)	11.3 ± 4.5 (10, 12.6)	9.8 ± 2.7 (8.8, 10.7)			
Missing	0 (0%)	1 (2%)	2 (4%)	2 (6%)			
**Psychological Distress**							
**Total BSI**					<0.001 * (d = 0.9)	0.297 (d = 0.3)	1.000 (d = 0)
Mean ± SD (95% CI)	18.8 ± 24.1 (11.9, 25.6)	45.9 ± 33.4 (37, 54.7)	27.2 ± 26.8 (19.6, 34.9)	18.6 ± 19.1 (11.7, 25.4)			
			1 (2%)	2 (6%)			
**Current Pain**							
**Pain VAS—During-CPT**					0.327 (d = 0.3)	0.629 (d = 0.3)	1.000 (d = 0.1)
Mean ± SD (95% CI)	48.2 ± 25.5 (40.9, 55.6)	56.3 ± 25.2 (49.5, 63.1)	54.7 ± 25.1 (47.4, 62)	50.7 ± 21.2 (42.9, 58.5)			
Missing	1 (2%)	2 (4%)	3 (6%)	3 (9%)			
**Pain VAS—Pre-CPT**					<0.001 * (d = 2.9)	<0.001 * (d = 2.2)	<0.001 * (d = 1.1)
Mean ± SD (95% CI)	5.1 ± 12.9 (1.4, 8.8)	66.6 ± 26.7 (59.6, 73.7)	53.6 ± 28.4 (45.5, 61.7)	26 ± 26.1 (16.5, 35.4)			
Missing			1 (2%)	2 (6%)			

**Table 3:** Comparison of clinical findings at admission, discharge, and follow-up for the treatment group. (N = number of subjects; s = seconds; SD = standard deviation; VAS = visual analog scale; FDI = functional disability inventory; PROMIS = patient reported outcomes information system anxiety assessment tool; GAD-7 = general anxiety disorder 7-item scale; BSI = brief symptom inventory; CPT = cold pressor test; * = statistically significant).

	Treatment—Admission (N = 57)	Treatment—Discharge (N = 51)	Treatment—Follow-Up (N = 34)	*p*-Values Admission vs. Discharge	*p*-Values Admission vs. Follow-Up	*p*-Values Discharge vs. Follow-Up
**Acute Pain**						
**Avg. Pain Threshold (s)**				1.000 (d = 0)	1.000 (d = −0.2)	1.000 (d = −0.2)
Mean ± SD (95% CI)	46.4 ± 39.7 (35.9, 56.9)	47.4 ± 51.1 (33.1, 61.8)	39.1 ± 36.2 (26.4, 51.7)			
**Avg. Pain Tolerance (s)**				1.000 (d = 0)	1.000 (d = 0.1)	1.000 (d = 0.1)
Mean ± SD (95% CI)	90.4 ± 62.9 (73.7, 107.1)	91.5 ± 68.3 (72.3, 110.7)	96.4 ± 70.9 (71.7, 121.1)			
**Sleep Impairment**						
**Sleep VAS Week**				1.000 (d = 0)	0.077 (d = −0.5)	0.140 (d = −0.5)
Mean ± SD (95% CI)	43 ± 28.3 (35.1, 51)	41.6 ± 27.8 (33.5, 49.8)	29.2 ± 25.6 (19.8, 38.6)			
Missing	6 (11%)	4 (8%)	3 (9%)			
**Sleep VAS Day**				0.743 (d = −0.2)	0.080 (d = −0.5)	0.712 (d = −0.3)
Mean ± SD (95% CI)	38.8 ± 29.8 (30.9, 46.7)	32.3 ± 28.4 (24.2, 40.4)	24.9 ± 26.6 (15.3, 34.5)			
Missing	0 (0%)	1 (2%)	2 (6%)			
**Functional Impairment**						
**FDI**				<0.001 * (d = −1.3)	<0.001 * (d = −1.6)	0.346 (d = −0.4)
Mean ± SD (95% CI)	24.5 ± 10.6 (21.6, 27.3)	11.9 ± 7.9 (9.7, 14.2)	8.8 ± 8.8 (5.7, 12)			
Missing	0 (0%)	1 (2%)	2 (6%)			
**Anxiety Symptomatology**						
**PROMIS**				0.013 * (d = −0.6)	<0.001 * (d = −0.8)	0.743 (d = −0.3)
Mean ± SD (95% CI)	19.9 ± 8.9 (17.5, 22.3)	15.3 ± 7.5 (13.1, 17.4)	13.5 ± 5.9 (11.4, 15.7)			
Missing	1 (2%)	1 (2%)	2 (6%)			
**GAD 7**				0.054 (d = −0.5)	<0.001 * (d = −0.8)	0.176 (d = −0.4)
Mean ± SD (95% CI)	13.7 ± 5.7 (12.2, 15.2)	11.3 ± 4.5 (10, 12.6)	9.8 ± 2.7 (8.8, 10.7)			
Missing	1 (2%)	2 (4%)	2 (6%)			
**Psychological Distress**						
**Total BSI**				0.005 * (d = −0.6)	<0.001 * (d = −0.9)	0.273 (d = −0.4)
Mean ± SD (95% CI)	45.9 ± 33.4 (37, 54.7)	27.2 ± 26.8 (19.6, 34.9)	18.6 ± 19.1 (11.7, 25.4)			
Missing	0 (0%)	1 (2%)	2 (6%)			
**Current Pain**						
**Pain VAS—During-CPT**				1.000 (d = −0.1)	0.840 (d = −0.2)	1.000 (d = −0.2)
Mean ± SD (95% CI)	56.3 ± 25.2 (49.5, 63.1)	54.7 ± 25.1 (47.4, 62)	50.7 ± 21.2 (42.9, 58.5)			
Missing	2 (4%)	3 (6%)	3 (9%)			
**Pain VAS—Pre-CPT**				0.051 (d = −0.5)	<0.001 * (d = −1.5)	<0.001 * (d = −1.0)
Mean ± SD (95% CI)	66.6 ± 26.7 (59.6, 73.7)	53.6 ± 28.4 (45.5, 61.7)	26 ± 26.1 (16.5, 35.4)			
Missing	0 (0%)	1 (2%)	2 (6%)			

**Table 4:** Linear mixed-effects model examining relationship between clinical factors in treatment group with admission as baseline. (VAS = visual analog scale; FDI = functional disability inventory; PROMIS = patient reported outcomes information system anxiety assessment tool; GAD-7 = general anxiety disorder 7-item scale; BSI = brief symptom inventory; CPT = cold pressor test; * = statistically significant).

Variable	Average Pain Threshold		Average Pain Tolerance	
	Estimate (SE)	*p*-Value	Estimate (SE)	*p*-Value
**Intercept**	7.53 (17.9)	0.677	24.51 (48.3)	0.614
**Age**	2.57 (1.1)	0.021 *	5.82 (3.0)	0.064
**Gender—Male**	6.66 (6.4)	0.305	17.18 (17.9)	0.343
**Discharge**	−2.45 (5.6)	0.664	−10.63 (11.6)	0.363
**Follow-up**	−1.77 (7.0)	0.800	−7.27 (14.6)	0.620
**Sleep Impairment**				
**Sleep VAS Week**	0.05 (0.1)	0.742	−0.04 (0.3)	0.900
**Sleep VAS Day**	−0.05 (0.1)	0.679	0.004 (0.2)	0.987
**Functional Impairment—FDI Score**	0.68 (0.3)	0.040 *	−0.03 (0.7)	0.967
**Anxiety Symptomatology**				
**PROMIS Score**	−0.71 (0.6)	0.251	0.54 (1.3)	0.682
**GAD 7**	0.19 (1.1)	0.860	−0.11 (0.4)	0.961
**Psychological Distress—Total BSI**	−0.04 (0.2)	0.800	−0.11 (0.4)	0.777
**Current Pain—Pain VAS Pre-CPT**	−0.20 (0.1)	0.055	−0.51 (0.2)	0.020 *

## Data Availability

The data underlying this article cannot be shared publicly due to the privacy of individuals that participated in the study. The data presented in this study are available on request from the corresponding author.

## References

[1] Weiss J.E., Stinson J.N. (2018). Pediatric Pain Syndromes and Noninflammatory Musculoskeletal Pain. Pediatr. Clin. N. Am..

[2] Chambers C.T., Dol J., Tutelman P.R., Langley C.L., Parker J.A., Cormier B.T., Macfarlane G.J., Jones G.T., Chapman D., Proudfoot N. (2024). The prevalence of chronic pain in children and adolescents: A systematic review update and meta-analysis. Pain.

[3] Bigatti S.M., Hernandez A.M., Cronan T.A., Rand K.L. (2008). Sleep disturbances in fibromyalgia syndrome: Relationship to pain and depression. Arthritis Rheum..

[4] Julien N., Goffaux P., Arsenault P., Marchand S. (2005). Widespread pain in fibromyalgia is related to a deficit of endogenous pain inhibition. Pain.

[5] Jensen K.B., Kosek E., Petzke F., Carville S., Fransson P., Marcus H., Williams S.C., Choy E., Giesecke T., Mainguy Y. (2009). Evidence of dysfunctional pain inhibition in Fibromyalgia reflected in rACC during provoked pain. Pain.

[6] Thastum M., Zachariae R., Scheler M., Bjerring P., Herlin T. (1997). Cold Pressor Pain: Comparing Responses of Juvenile Arthritis Patients and Their Parents. Scand. J. Rheumatol..

[7] Tsao J.C., Evans S., Seidman L.C., Zeltzer L.K. (2012). Experimental pain responses in children with chronic pain and in healthy children: How do they differ?. Pain Res. Manag..

[8] Paul-Savoie E., Marchand S., Morin M., Bourgault P., Brissette N., Rattanavong V., Cloutier C., Bissonnette A., Potvin S. (2012). Is the deficit in pain inhibition in fibromyalgia influenced by sleep impairments?. Open Rheumatol. J..

[9] Stevens A., Batra A., Kötter I., Bartels M., Schwarz J. (2000). Both pain and EEG response to cold pressor stimulation occurs faster in fibromyalgia patients than in control subjects. Psychiatry Res..

[10] Palermo T.M., Kiska R. (2005). Subjective sleep disturbances in adolescents with chronic pain: Relationship to daily functioning and quality of life. J. Pain.

[11] Olsen M.N., Sherry D.D., Boyne K., McCue R., Gallagher P.R., Brooks L.J. (2013). Relationship between sleep and pain in adolescents with juvenile primary fibromyalgia syndrome. Sleep.

[12] McGrath P.J., Walco G.A., Turk D.C., Dworkin R.H., Brown M.T., Davidson K., Eccleston C., Finley G.A., Goldschneider K., Haverkos L. (2008). Core outcome domains and measures for pediatric acute and chronic/recurrent pain clinical trials: PedIMMPACT recommendations. J. Pain.

[13] Gmuca S., Sherry D.D. (2017). Fibromyalgia: Treating Pain in the Juvenile Patient. Paediatr. Drugs.

[14] Harrison L.E., Pate J.W., Richardson P.A., Ickmans K., Wicksell R.K., Simons L.E. (2019). Best-Evidence for the Rehabilitation of Chronic Pain Part 1: Pediatric Pain. J. Clin. Med..

[15] Sherry D.D., Brake L., Tress J.L., Sherker J., Fash K., Ferry K., Weiss P.F. (2015). The Treatment of Juvenile Fibromyalgia with an Intensive Physical and Psychosocial Program. J. Pediatr..

[16] Cazzola M., Atzeni F., Boccassini L., Cassisi G., Sarzi-Puttini P. (2014). Physiopathology of pain in rheumatology. Reumatismo.

[17] Liossi C., Johnstone L., Lilley S., Caes L., Williams G., Schoth D.E. (2019). Effectiveness of interdisciplinary interventions in paediatric chronic pain management: A systematic review and subset meta-analysis. Br. J. Anaesth..

[18] Sherry D.D., Sonagra M., Gmuca S. (2020). The spectrum of pediatric amplified musculoskeletal pain syndrome. Pediatr. Rheumatol. Online J..

[19] Alqurashi Y.D., Dawidziuk A., Alqarni A., Kelly J., Moss J., Polkey M.I., Morrell M.J. (2021). A visual analog scale for the assessment of mild sleepiness in patients with obstructive sleep apnea and healthy participants. Ann. Thorac. Med..

[20] Jensen M.P., Karoly P., Braver S. (1986). The measurement of clinical pain intensity: A comparison of six methods. Pain.

[21] Walker L.S., Greene J.W. (1991). The functional disability inventory: Measuring a neglected dimension of child health status. J. Pediatr. Psychol..

[22] Kashikar-Zuck S., Flowers S.R., Claar R.L., Guite J.W., Logan D.E., Lynch-Jordan A.M., Palermo T.M., Wilson A.C. (2011). Clinical utility and validity of the Functional Disability Inventory among a multicenter sample of youth with chronic pain. Pain.

[23] Claar R.L., Walker L.S. (2006). Functional assessment of pediatric pain patients: Psychometric properties of the functional disability inventory. Pain.

[24] Derogatis L.R. (1993). BSI Brief Symptom Inventory: Administration, Scoring, and Procedures Manual.

[25] Conoley J.C., Kramer J.J. (1989). The Tenth Mental Measurements Yearbook.

[26] Kroenke K., Spitzer R.L., Williams J.B.W., Löwe B. (2010). The Patient Health Questionnaire Somatic, Anxiety, and Depressive Symptom Scales: A systematic review. Gen. Hosp. Psychiatry.

[27] Mossman S.A., Luft M.J., Schroeder H.K., Varney S.T., Fleck D.E., Barzman D.H., Gilman R., DelBello M.P., Strawn J.R. (2017). The Generalized Anxiety Disorder 7-item scale in adolescents with generalized anxiety disorder: Signal detection and validation. Ann. Clin. Psychiatry Off. J. Am. Acad. Clin. Psychiatr..

[28] Spitzer R.L., Kroenke K., Williams J.B., Löwe B. (2006). A brief measure for assessing generalized anxiety disorder: The GAD-7. Arch. Intern. Med..

[29] Pilkonis P.A., Choi S.W., Reise S.P., Stover A.M., Riley W.T., Cella D., PROMIS Cooperative Group (2011). Item banks for measuring emotional distress from the Patient-Reported Outcomes Measurement Information System (PROMIS^®^): Depression, anxiety, and anger. Assessment.

[30] von Baeyer C.L., Piira T., Chambers C.T., Trapanotto M., Zeltzer L.K. (2005). Guidelines for the cold pressor task as an experimental pain stimulus for use with children. J. Pain.

[31] Liossi C., Laycock H., Radhakrishnan K., Hussain Z., Schoth D.E. (2024). A Systematic Review and Meta-Analysis of Conditioned Pain Modulation in Children and Young People with Chronic Pain. Children.

[32] Birnie K.A., Petter M., Boerner K.E., Noel M., Chambers C.T. (2012). Contemporary Use of the Cold Pressor Task in Pediatric Pain Research: A Systematic Review of Methods. J. Pain.

[33] Birnie K.A., Noel M., Chambers C.T., von Baeyer C.L., Fernandez C.V. (2011). The cold pressor task: Is it an ethically acceptable pain research method in children?. J. Pediatr. Psychol..

